# Construction of a Comprehensive Multiomics Map of Hepatocellular Carcinoma and Screening of Possible Driver Genes

**DOI:** 10.3389/fgene.2020.00634

**Published:** 2020-06-25

**Authors:** Ziyu Liu, Yan Lin, Xing Gao, Rongyun Mai, Xuemin Piao, Jiazhou Ye, Rong Liang

**Affiliations:** ^1^Department of Medical Oncology, Guangxi Medical University Cancer Hospital, Nanning, China; ^2^Department of Hepatobiliary Surgery, Guangxi Medical University Cancer Hospital, Nanning, China

**Keywords:** hepatocellular carcinoma, driver genes, methylation, multiomics, mutational signatures, APOBEC

## Abstract

**Objectives:** The occurrence of hepatocellular carcinoma (HCC) is a complex process involving genetic mutations, epigenetic variation, and abnormal gene expression. However, a comprehensive multiomics investigation of HCC is lacking, and the available multiomics evidence has not led to improvements in clinical practice. Therefore, we explored the molecular mechanism underlying the development of HCC through an integrative analysis of multiomics data obtained at multiple levels to provide innovative perspectives and a new theoretical basis for the early diagnosis, personalized treatment and medical guidance of HCC.

**Methods:** In this study, we collected whole-exome sequencing data, RNA (mRNA and miRNA) sequencing data, DNA methylation array data, and single nucleotide polymorphism (SNP) array data from The Cancer Genome Atlas (TCGA). We analyzed the copy number variation (CNV) in HCC using GISTIC2. MutSigCV was applied to identify significantly mutated genes (SMGs). Functional enrichment analyses were performed using the clusterProfiler package in R software. The prognostic values of discrete variables were estimated using Kaplan–Meier survival curves.

**Results:** By analyzing the HCC data in TCGA, we constructed a comprehensive multiomics map of HCC. Through copy number analysis, we identified significant amplification at 29 loci and significant deletions at 33 loci. A total of 13 significant mutant genes were identified. In addition, we also identified three HCC-related mutant signatures, and among these, signature 22 was closely related to exposure to aristolochic acids. Subsequently, we analyzed the methylation level of HCC samples and identified 51 epigenetically silenced genes that were significantly associated with methylation. The differential expression analysis identified differentially expressed mRNAs and miRNAs in HCC samples. Based on the above-described results, we identified a total of 93 possible HCC driver genes, which are driven by mutations, methylation, and CNVs and have prognostic value.

**Conclusion:** Our study reveals variations in different dimensions of HCC. We performed an integrative analysis of genomic signatures, single nucleotide variants (SNVs), CNVs, methylation, and gene expression in HCC. Based on the results, we identified HCC possible driver genes that might facilitate prognostic prediction and support decision making with regard to the choice of therapy.

## Introduction

Hepatocellular carcinoma (HCC) is a leading cause of cancer-related death in many parts of the world, and its surveillance and early detection increase the possibility of potentially curative treatment (Llovet et al., 2018). There are few effective treatments for HCC, and no mutations that are targeted by available drugs have been identified in HCC patients (Liu et al., 2019). Intratumoral heterogeneity is one of the main reasons for the ineffectiveness of the current therapies in most types of cancer, including HCC (Zhang et al., 2019). Thus, the mechanism of HCC development needs to be elucidated, and the identification of effective prognostic molecular markers is of great significance for the individualized diagnosis and treatment of HCC.

Whole-genome and exome sequencing studies conducted in recent years have revealed the mutational landscape of HCC (Nie et al., 2014; Fujimoto et al., 2016), and omics analyses of HCC have broadened our knowledge of the molecular events related to this fatal malignancy. Li et al. performed an integrative analysis of 1,061 HCC genomes and identified 11 novel mutant genes (Li et al., 2018). Pan et al. sequenced the transcriptome of HCC patients and identified 755 differentially expressed genes (DEGs). These researchers also identified 15 hub genes in the module associated with the alpha fetoprotein (AFP) level (Pan et al., 2016). Cheng et al. identified a group of patients with a CpG island methylator phenotype (CIMP) and found that the overall survival (OS) rate of CIMP patients was poorer than that of non-CIMP patients. These researches also identified promising biomarkers for the diagnosis of HCC (Cheng et al., 2018). However, these above-mentioned studies focused on single genes or individual omics, and conducting an in-depth systematic study of the molecular mechanism of HCC from a comprehensive, multidimensional perspective is thus difficult.

The development of HCC is a complex biological process that involves the interaction of multiple omics, and thus, a comprehensive multiomics analysis of the variation in HCC can accelerate our understanding of disease development and provide a new and effective solution for clinical diagnosis and treatment (Miao et al., 2014; Large et al., 2017). At present, international research on malignant tumors has been performed at the genomic, transcriptomic, and epigenetic levels using high-throughput sequencing technology (Chen et al., 2017; Ortega et al., 2017; Bareche et al., 2018). The obtained data have been combined with clinical information to reveal the occurrence and development of malignant tumors. The molecular mechanisms provide a basis for the identification of effective therapeutic targets and the development of personalized treatment strategies. However, multiomics research on HCC is scarce.

The integration of multiple levels of omics data is important research direction for obtaining a comprehensive and systematic understanding of HCC. Therefore, we conducted a comprehensive analysis of the HCC data included in The Cancer Genome Atlas (TCGA). By combined these data with clinical information, this study aimed to comprehensively clarify the role of different levels of variability in HCC. Driver genes are directly responsible for carcinogenesis and are closely related to the development of cancer (Dietlein et al., 2020). A major goal of cancer genomics is to identify these rare driver genes amid the myriad passengers (Merid et al., 2014). Cancer driver genes by definition carry at least one driver mutations that increase cell growth advantage (Nono et al., 2019). Therefore, combined with multi-omics analysis, we identified possible driver genes in HCC samples, and these findings lay a theoretical foundation for the prevention, individualized treatment, and even exploration of potential therapeutic targets of HCC.

## Materials and Methods

**Figure 1:**
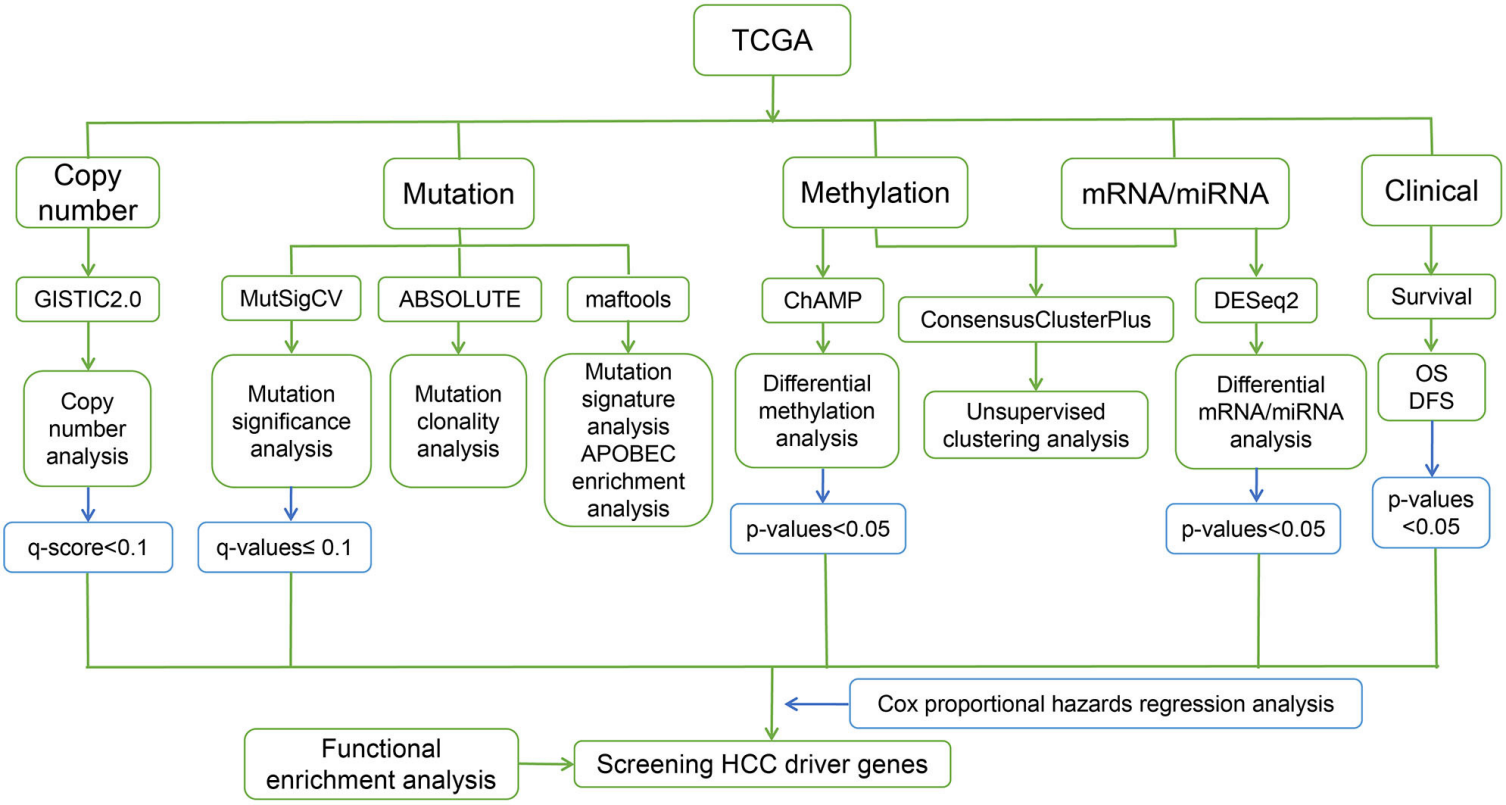
Workflow of the present study.

The steps of the workflow used in this study are shown in Figure 1.

### Datasets

MalaCards (http://www.malacards.org/) was used to explore the genes associated with HCC. The RNA-seq data, methylation data, mutation data, and corresponding clinical information for HCC patients were obtained from TCGA (https://tcga-data.nci.nih.gov/tcga/) cohort (Tomczak et al., 2015). We accessed TCGA and downloaded whole-exome sequencing data, RNA (mRNA and miRNA) sequencing data, DNA methylation array data, and single nucleotide polymorphism (SNP) array data. We also downloaded the clinical information of HCC patients in TCGA for subsequent analysis.

### Copy Number Analysis

The Genetic Identification of Significant Targets in Cancer version 2.0 (GISTIC2.0) program (Mermel et al., 2011) was used to identify significant regions showing broad and focal amplification and deletion across all the samples. The copy number values were obtained by examining the distribution of log2 ratios to identify peaks associated with copy number states, and q-values based on permutations of copy number segment locations were used to assess the significance of G-scores. A *q*-score of < 0.1 and a confidence interval of 95% were considered to determine significance. The default GISTIC threshold for identifying gains and losses was used. The GISTIC algorithm considers both high and low thresholds for copy number determination across all the input samples to assign significance to CNVs. The copy number statuses of low-level gene amplification, high-level gene amplification, low-level gene deletion, and high-level gene deletion was inferred using the “thresholded” calls.

### Identification of Significantly Mutated Genes (SMGs)

The mutational data were saved in the mutation annotation format (maf), and the maf data were processed using the R package maftools (Mayakonda et al., 2018). The mutation spectra were analyzed by non-negative matrix factorization (NMF). The maf mutation file was analyzed using MutSigCV (version 1.3.4) (Lawrence et al., 2013) to recognize significant SMGs based on the significance threshold. MutSigCV quantifies the significance of non-silent mutations in a gene based on the background mutation rate estimated by silent mutations, and other confounding covariates are also considered in this analysis. The significance levels (*p*-values) were determined by testing whether the observed mutations in a gene significantly exceeded the expected counts based on the background model. The false discovery rates (*q*-values) were then calculated, and genes with *q*-values ≤ 0.1 were regarded as significantly mutated. We then utilized maftools to visualize the mutation information of these significant SMGs among HCC patients in TCGA.

### DNA Methylation Analysis

After downloading DNA methylation data of HCC patients in TCGA, we performed further analyses using the Chip Analysis Methylation Pipeline (ChAMP) package (Morris et al., 2013) in R language. The DNA methylation value was calculated from the intensity data files using the ChAMP package in the R programming language and was quantified as a beta-value. The ChAMP program was run using the default parameter settings, and this program first subjects the data to a number of quality control (QC) filtering steps, which included probes with poor removal results, multiple hit probes, probes with overlapping genomic regions, and probes within the XY chromosome. After QC, the remaining probes were utilized to identify differentially methylated probes (DMPs) and differentially methylated regions (DMRs) between cancer-paracancer using the ChAMP R pipeline with a linear model. The *p* < 0.05 was considered to be significantly different.

### Identification and Scoring of Differentially Expressed Genes in HCC

We obtained 50 pairs of RNA sequencing data and analyzed differential expression patterns between cancer and paracancerous tissues in TCGA. The R language package DEseq2 (Love et al., 2014) was used to identify differentially expressed mRNAs (DEmRNAs) and miRNAs (DEmiRNAs) between HCC and normal liver tissue from mRNA/miRNA sequencing data of HCC obtained from TCGA. The fold changes (FCs) in the expression of individual genes were calculated, and genes with |log2FC| > 1 and *p* < 0.05 adjusted by the false discovery rate (FDR) were considered significant. Differentially expressed volcano maps were generated using the ggplot2 package (Ginestet, 2011) of R software.

We then screened differentially expressed genes with |log2FC| > 1 and *p* < 0.05, and the changes in the expression levels of these genes were consistent with the changes in the hazard ratios (HRs) of overall survival (OS) and disease-free survival (DFS). We used regression analysis to score each gene and calculate the *p*-value.

### Unsupervised Clustering Analysis

The ConsensusClusterPlus package (Wilkerson and Hayes, 2010) was utilized to perform consistent clustering analyses using K-means. The clustering was performed using 100 iterations, and each iteration contained 80% of the samples. The optimal cluster number was determined by the cumulative distribution function (CDF) curves of the consensus score, and the heatmap was then constructed using the pheatmap package (Quackenbush, 2011) in R software.

### Functional Enrichment Analyses of Driver Genes

To explore the biology of the gene modules, Gene Ontology (GO) and Kyoto Encyclopedia of Genes and Genomes (KEGG) pathway enrichment analyses were performed using the clusterProfiler package (Yu et al., 2012) in R software. The *p*-value was adjusted using the Benjamini and Hochberg algorithm, and *p* < 0.05 was considered to indicate statistical significance. The figure was drawn using the ggplot2 package (Ginestet, 2011) in R software.

### Survival Analysis

The prognostic values of discrete variables were estimated by Kaplan–Meier survival curves (Bland and Altman, 1998), and the log-rank test was employed to estimate significant differences between survival curves. Univariate and multivariate Cox models were constructed to estimate the HRs of the prognosticators with a *p* < 0.05 in the log-rank test. Various outcomes, including OS and DFS, were investigated. All the statistical analyses were performed using the R survival package with the default parameters.

## Results

### Copy Number Alterations (CNAs) in HCC

**Figure 2:**
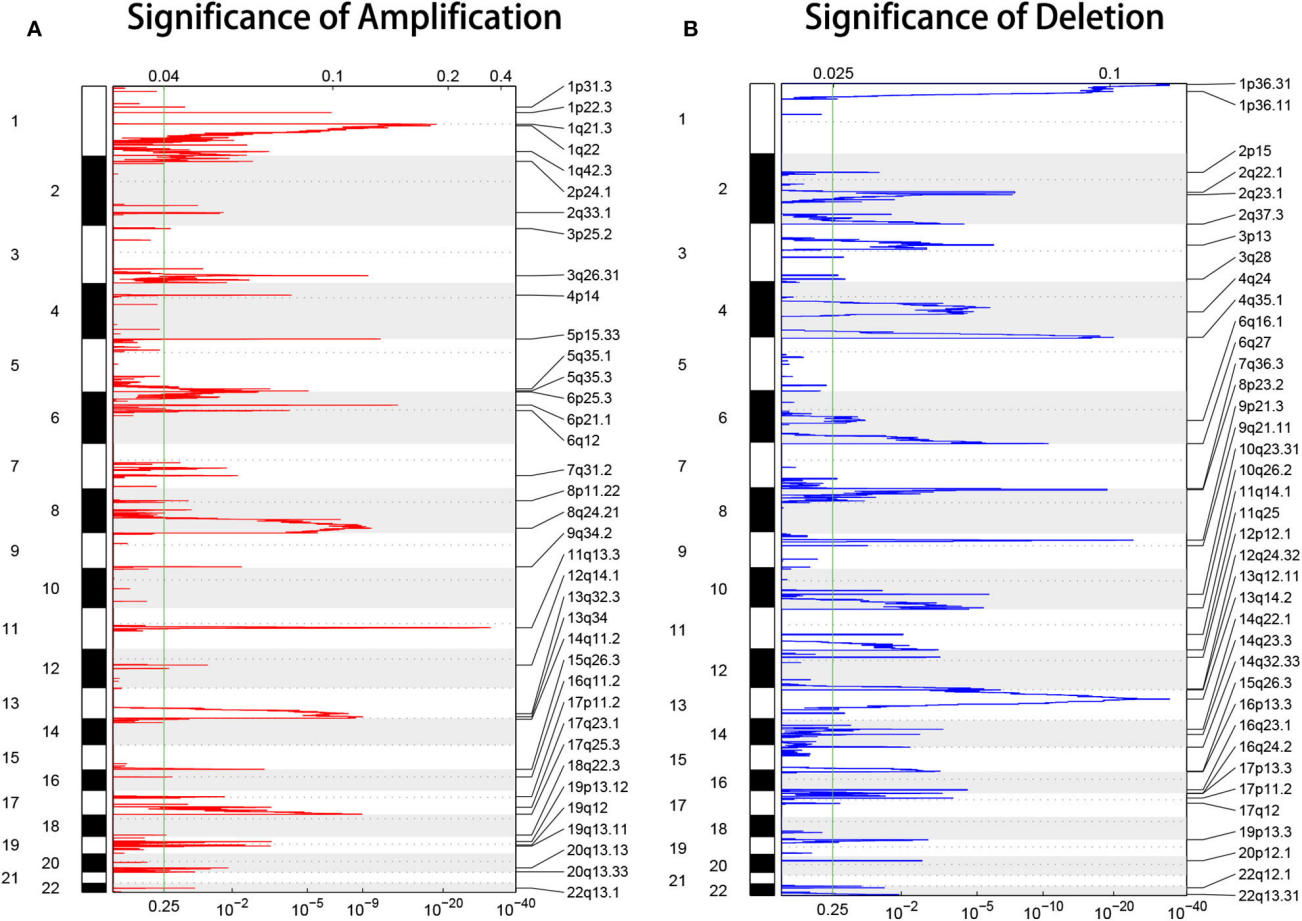
GISTIC heat map showing the genomic copy number profiles from the LIHC cohort in TCGA. **(A,B)** The gain (red) and loss (blue) of each peak are shown. The x-axes represent the normalized amplification signal (top) and the significance of the *q*-value (bottom). The green line represents the significance cutoff at a *q*-value of 0.25.

To investigate the molecular basis of HCC progression and explore new molecular diagnostic approaches, DNA CNAs were analyzed, and genome-wide focal DNA gains, and losses were delineated. Significant arm-level alterations included the gain of 1q, 8q, 5p, 6p, 5q, 7p, 7q, and 20q (Figure 2A) and the loss of 17p, 8p, 16q, 13q, 14q, 9p, and 9q (Figure 2B). We identified significant amplifications at 29 loci and significant deletions at 33 loci (*p* < 0.05). As expected, our analysis confirmed many known CNAs, including those observed at 11q13.3 (CCND1), 6p21.1 (VEGFA), 5p15.33 (TERT), and 8q24.21 (MYC), and focal deletions, including those observed at 9p21.3 (CDKN2A), 13q14.2 (RB1), and 10q23.31 (PTEN). Collectively, these data reveal the CNA environment in HCC and provide insights for subsequent research on HCC. The tumor ploidy estimated by ABSOLUTE (Carter et al., 2012) revealed that a large proportion of HCCs exhibited genome doubling (Table S1).

### Significantly Mutated Genes in HCC

**Figure 3:**
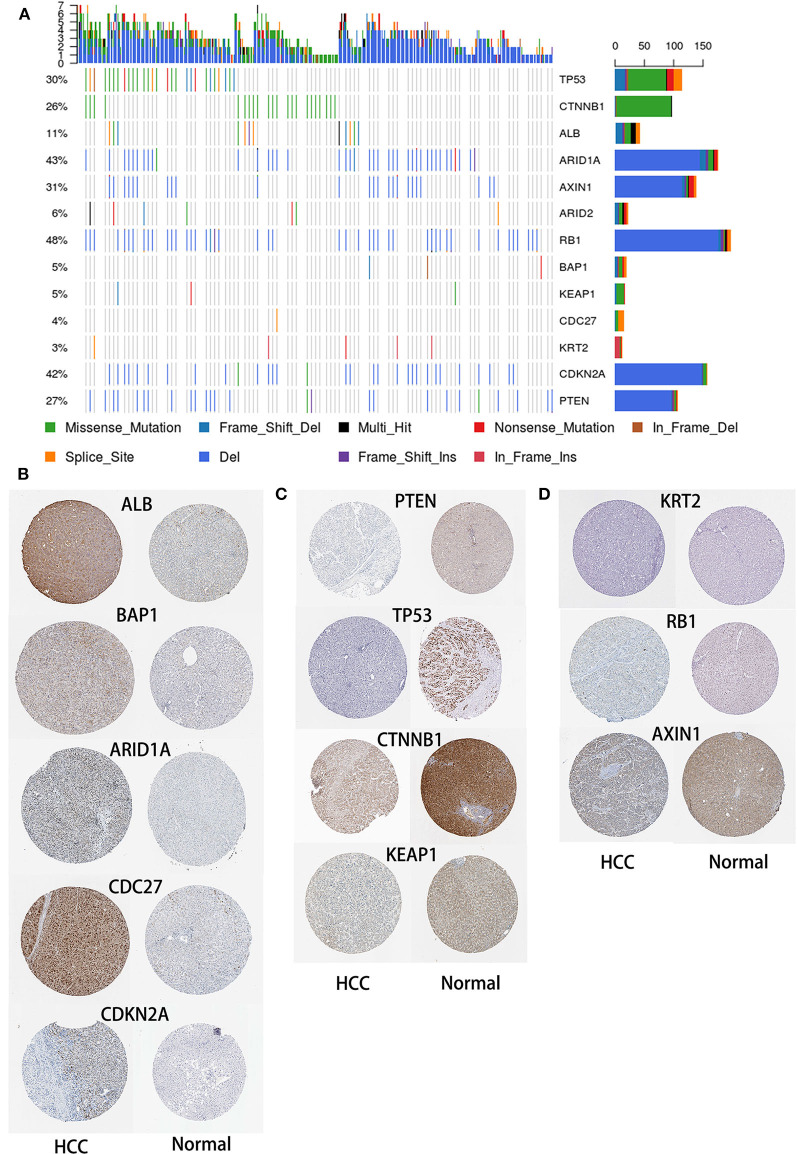
Mutational landscape of SMGs in HCC. **(A)** Significantly mutated genes in HCC. The different colors represent different mutation types. **(B)** Expression of SMGs in HCC and normal tissues. According to an analysis of immunohistochemical staining data from the Human Protein Atlas database, the expression of SMGs in HCC was compared with that in normal tissues.

To identify SMGs associated with HCC development, we identified genes whose mutations were positively accumulated, clustered at a hotspot, and of functional importance. In total, we identified 13 SMGs (*q* < 0.01). Most of these SMGs, such as TP53, CTNNB1, and BAP1, have been identified in previous studies. In addition, we also identified some novel SMGs, such as CDC27, CDKN2E, KRT2, and ALB, which have rarely been detected in HCC research (Figure 3A).

### Mutational Signatures Operative in HCC

**Figure 4:**
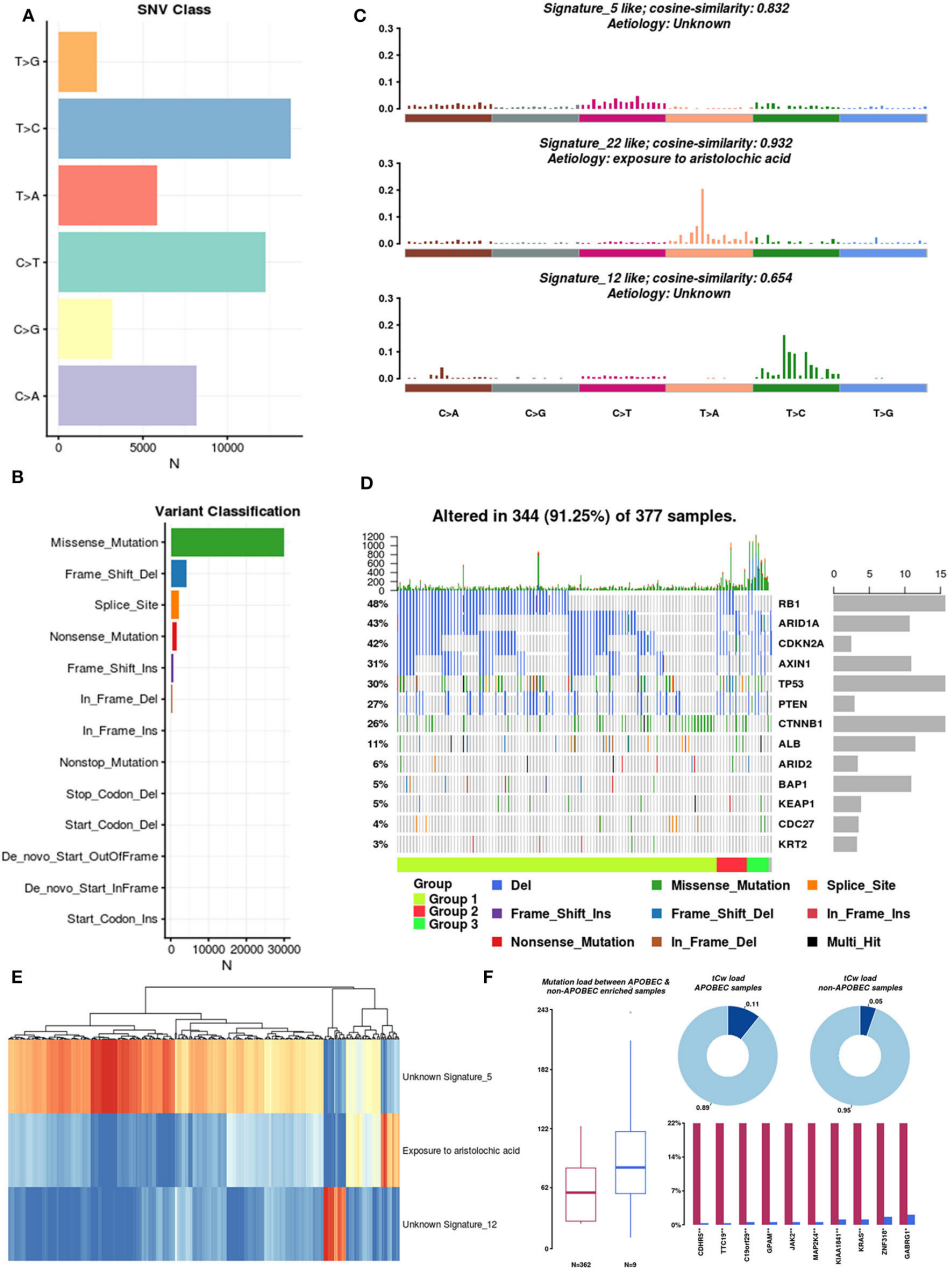
Mutation signatures operative in HCC. **(A)** Single nucleotide variation classification: different colors represent different types of variation. **(B)** Variant classification: different colors represent different variant classifications. **(C)** Mutational signatures: spectrum of total SNVs and three mutational signatures in the context of the 96 base-pair substitutions. Because the dynamic range for the signatures is large, the upper limits of the y-axis used for each signature are different. **(D)** SMG mutation rate in three groups of HCC samples. **(E)** Unsupervised clustering of mutations in SMGs and focal SCNAs. **(F)** Differences in mutational patterns between APOBEC-enriched and non-APOBEC-enriched samples.

There are six types of single nucleotide variations (SNVs), and among these, T>C is dominant (Figure 4A). Regardless of the sample or the frequently mutated gene, missense mutations accounted for the largest proportion (Figure 4B). In the context of the 96 base-pair substitutions, we analyzed the spectrum of total SNVs and identified three mutational signatures (signatures 5, 22, and 12) (Figure 4C). The etiology of signature 22 was closely related to exposure to aristolochic acids (AAs). Chinese herbal medicines containing AAs were recently reported as contributors to oncogenesis, including HCC oncogenesis (Ng et al., 2017). Based on these three mutational signatures, we divided HCC patients into three groups and analyzed the mutation rates of 13 SMGs in each group (Figure 4D). Subsequently, we clustered the mutations in the SMGs and focal somatic copy number alterations (SCNAs). The results showed that these three mutation signatures can be used to classify HCC samples (Figure 4E).

Pan-cancer mutational signature analyses have identified a signature consisting of apolipoprotein B mRNA editing enzyme, catalytic polypeptide-like (APOBEC), which is a cytosine deaminase, in a subset of cancers, including HCC (Middlebrooks et al., 2016). We integrated a method described by Roberts et al. (2013) to estimate the enrichment of APOBEC in individual tumor samples. A total of 371 samples were included in the analysis, and 97.6% (362 of 371 samples) of HCC samples were enriched in APOBEC-associated mutations (APOBEC enrichment score > 2). The mutation burdens among non-APOBEC samples were significantly higher than those among APOBEC-enriched samples. Furthermore, on the tCw background, the APOBEC-enriched samples exhibited higher mutation frequencies than the non-APOBEC-enriched samples (0.11 vs. 0.05, respectively) (Figure 4F). Of interest, we identified the top 10 differentially mutated genes between APOBEC-enriched and non-APOBEC-enriched samples, and among these, KRAS, CDHR5, JAK2, and MAP2K4 were previously shown to be closely related to tumor development. Overall, APOBEC enrichment is strongly associated with the mutational signature in HCC.

### DNA Methylation and Epigenetically Silenced Genes in HCC

**Figure 5:**
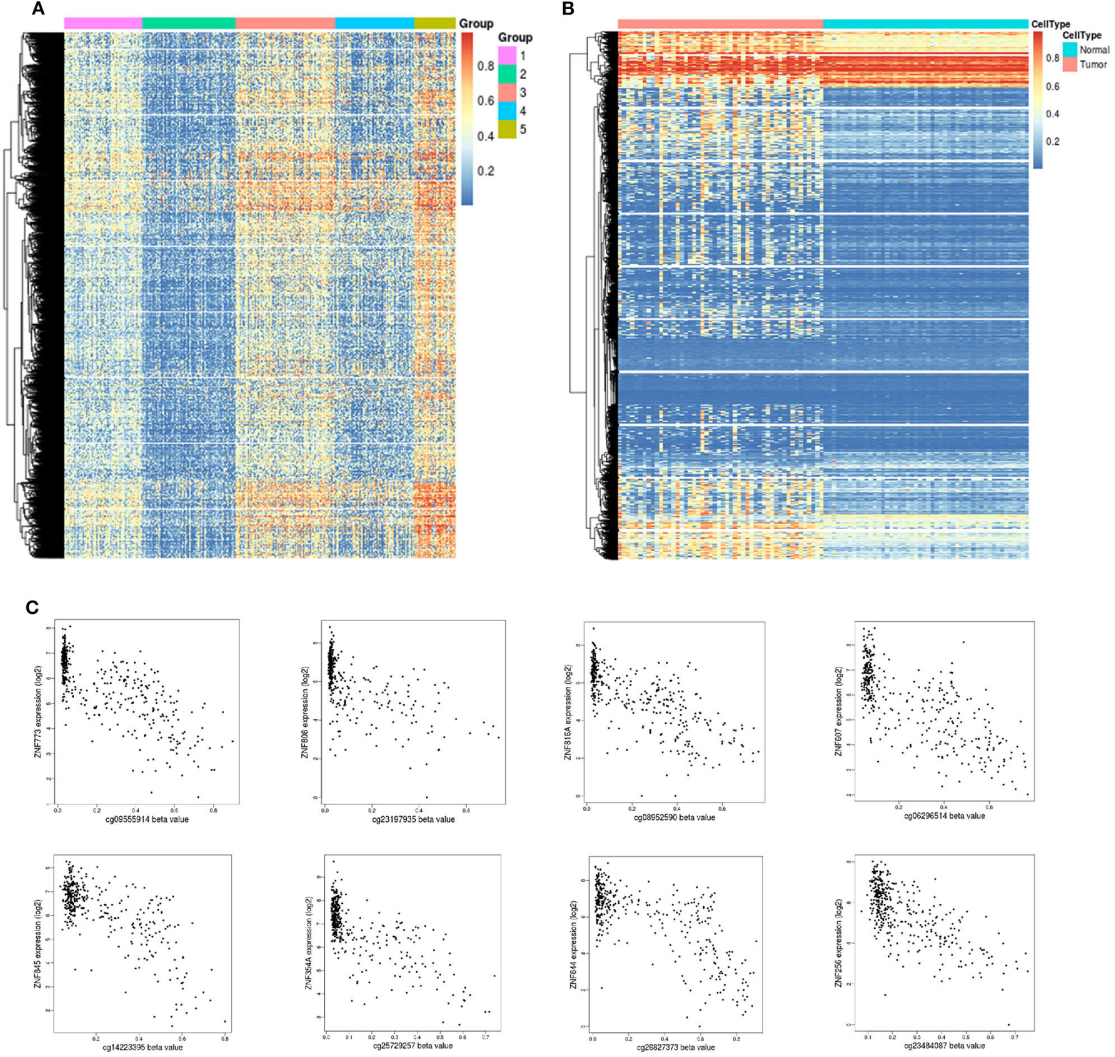
Unsupervised clustering analysis of DNA methylation. **(A)** Heat map and unsupervised hierarchical clustering of DNA methylation profiles. The different colored bar charts represent different groups. Hypermethylation is marked in red, and hypomethylation is marked in blue. **(B)** Heat map and unsupervised hierarchical clustering of methylation differences between tumor and normal tissues. The samples under the red bar represent normal samples, and the samples under the blue bar represent HCC samples. Hypermethylation is marked in red, and hypomethylation is marked in blue. **(C)** Epigenetically silenced genes.

DNA methylation is an early event in tumorigenesis. Here, to advance our understanding of DNA methylation in HCC, all methylated samples were divided into different k groups. Based on the CDF curves of the consensus score, optimal division was reached with a *k* value of 5 (Figure 5A). Notably, group 2 exhibited a low level of methylation, whereas group 5 had a high methylation level. To distinguish differences in the methylation profiles between tumor and paracancerous tissues, we systematically analyzed their methylation profiles, and the results showed that tumor samples exhibited higher methylation levels than paracancerous samples (Figure 5B).

Promoter methylation might cooperate with loss (gene silencing) and enhances the malignant phenotype associated with chromosomal aberrations or promotes chromosomal instability and loss of the unaffected allele. DNA methylation is a repressive marker critical for epigenetic gene silencing. Based on methylation data in TCGA, we identified 51 significantly silenced genes (|Cor| > 0.7) (Table S2), and interestingly, 10 genes from the zinc finger protein (ZNF) family were included in this sett (Figure 5C). The vast majority of ZNF functions include interaction modules that bind DNA, RNA, proteins, or other small, useful molecules, and structural variations serve primarily to alter the binding specificity of a particular protein and might be involved in transcriptional regulation (Klug and Rhodes, 1987).

### Analysis of Differentially Expressed Genes in HCC

**Figure 6:**
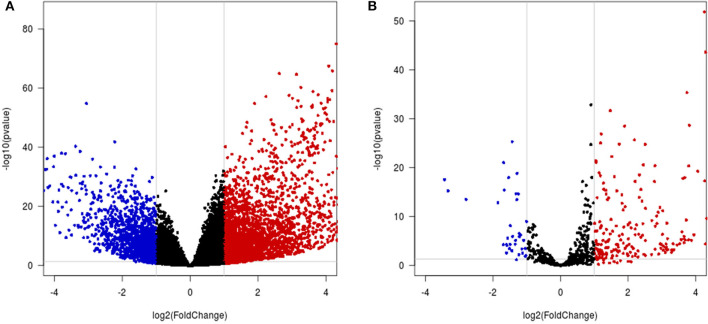
Differential mRNA and miRNA expression analysis. **(A,B)** Volcano map of differentially expressed mRNAs/miRNAs. The x-axis indicates the fold-change, and the y-axis indicates the negative logarithm base 10 of the adjusted p-value. The gray vertical and horizontal lines reflect the filtering criteria. The red and blue dots represent the significantly upregulated and downregulated genes, respectively.

A total of 4,065 DEmRNAs, which included 2,727 upregulated and 1,338 downregulated DEmRNAs, were identified in the HCC samples compared with the control samples (Figure 6A, Table S3). A total of 228 DEmiRNAs were identified in the HCC tissues compared with the control samples, and these included 192 upregulated and 36 downregulated DEmiRNAs (Figure 6B, Table S4).

The top 10 most significant DEmRNAs were GABRD, THBS4, COL15A1, CDKN3, KIF4A, SLC26A6, PLVAP, CDCA5, KIF2C, and KIFC1, and subsequent analysis of these genes revealed that some play an important role in HCC. THBS4 enhances HCC migration and vascular invasion (Su et al., 2017). Wu et al. (2019) identified CDKN3 in HCC at very early stages. CDKN3 is considered a key gene for HCC initiation, and according to a study conducted by Hu, KIF4A promotes HCC cell proliferation (Hu et al., 2019). In addition, CDCA5 regulates proliferation in HCC and might serve as a negative prognostic marker (Shen et al., 2018). Although the mechanisms of the other genes in HCC have not been extensively explored, their function has been verified in other tumors. Whether these genes play the same role in HCC is worthy of further experimental research.

In addition, we scored the differentially expressed genes through regression analyses. A larger score indicates that the gene is more closely related to the development of HCC. A gene that is highly expressed in high-risk cancer samples, as assessed by an analysis of OS or DFS, can be considered a likely oncogene, and low expression in these samples could indicate a tumor suppressor. A total of 341 genes, including 15 genes with a score of 1.5–2.0, 290 genes with a score of 1.0–1.5, and 37 genes with a score of 0.5–1.0, were screened (Table S5). The gene with the highest score was SPRYD4, and Zahid et al. (2019) found that the SPRYD4 gene was downregulated in HCC tissues compared with non-tumor tissues and that exogenous SPRYD4 expression inhibits HCC cell proliferation by inducing apoptosis.

### Identification of HCC Possible Driver Genes

Identifying cancer driver genes is a crucial step in cancer genomic toward the advancement of precision medicine. The accumulation of alterations in cancer possible driver genes is a major trigger for hepatocarcinogenesis and tumor progression, and their identification is thus essential for obtaining a full understanding of the mechanisms of cancer.

**Figure 7:**
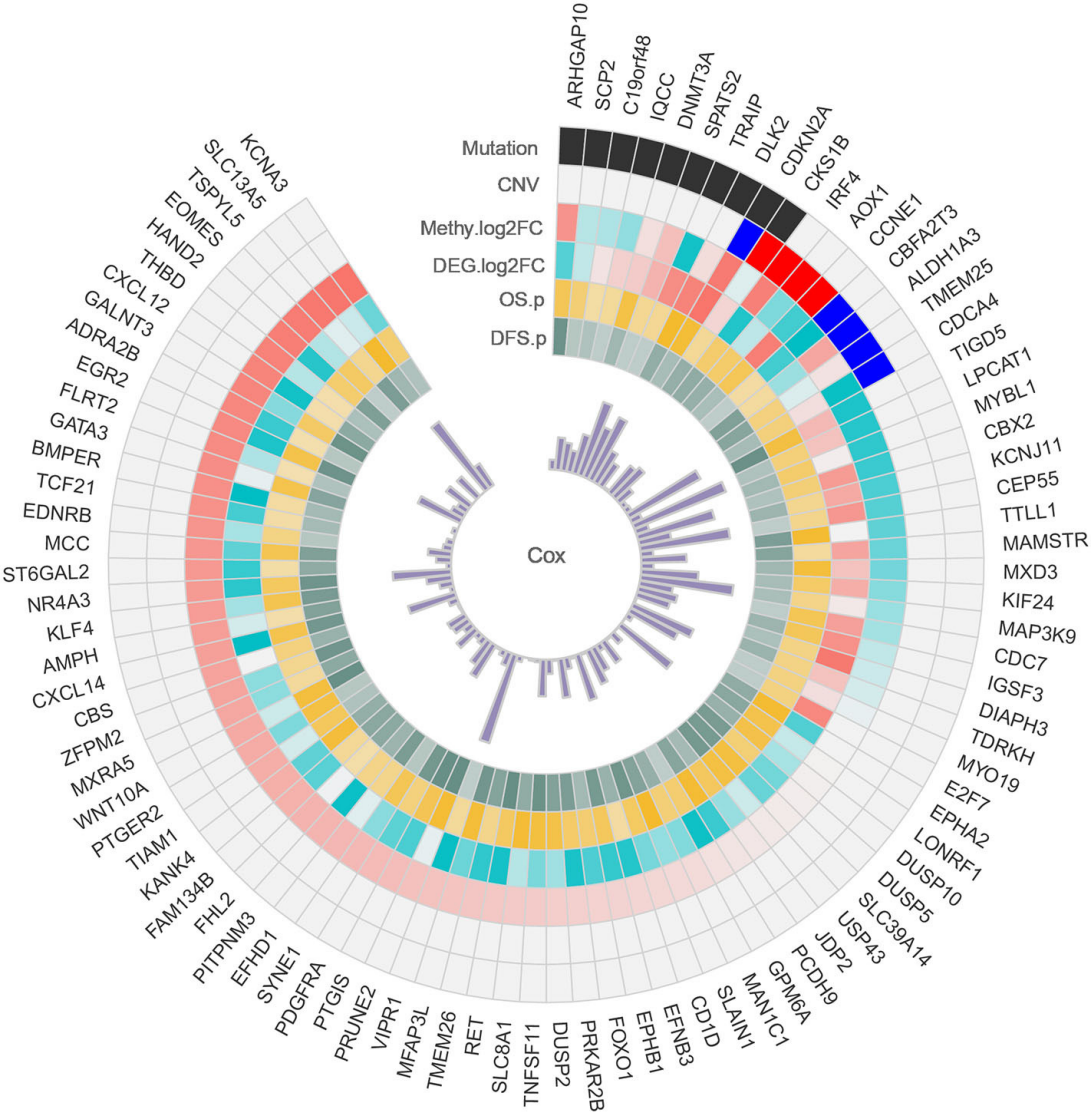
Circos plot of possible driver genes. Each circle represents a gene driven by a mutation, CNV, methylation, and differential expression.

Alterations in the genetic makeup of a cell, such as mutation, CNV and methylation, can all lead to the development of cancer (Hanahan and Weinberg, 2011). CNV promotes tumorigenesis and progression by activating oncogenes or inactivating tumor suppressor genes (Zack et al., 2013). Disruption of epigenetic mechanisms can lead to hypermethylation or hypomethylation of gene promoter regions and may lead to the silencing of key tumor suppressor functions (Kulis and Esteller, 2010). Discovery mutated driver genes from passenger mutations is one of the primary task in tumorigenesis, and driver gene mutations promote cancer progression, and have major impacts on patient outcome (Vogelstein et al., 2013). When a gene is variated at multi-omics levels, we have reason to believe that it plays an important role in the development of cancer. Therefore, we conducted a comprehensive analysis of HCC multi-omics, and identified some important genes in each omics. Combined with multi-omics analysis, we identified a total of 93 possible driver genes in HCC (Figure 7), and all of these genes were methylated genes that are differentially expressed in HCC and have prognostic significance. Among these genes, 10 genes are driven by mutations, and nine genes are driven by CNVs. In particular, CDKN2A and CKS1B are driven by both mutations and CNVs. Collectively, these data identify important genes involved in the tumorigenesis of HCC.

### Functional Enrichment Analysis of Driver Genes

**Figure 8:**
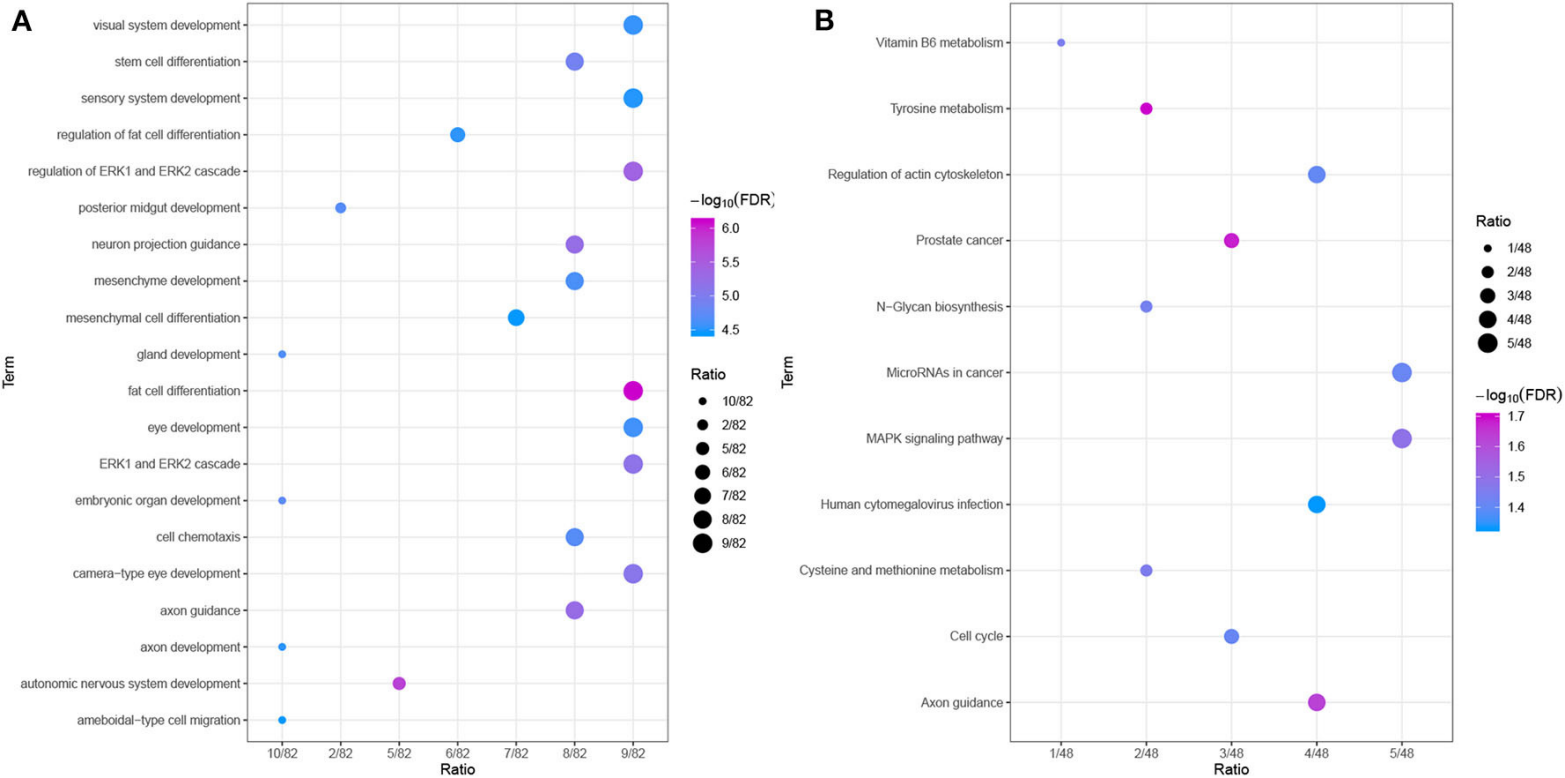
Functional enrichment analyses. **(A)** GO analysis of the possible driver genes. The possible driver genes are significantly enriched in 20 biological processes. **(B)** KEGG pathway analysis of the possible driver genes. The possible driver genes are significantly enriched in 11 KEGG pathways.

To further explore the relevant functions of the identified possible driver genes, we performed GO and KEGG pathway analyses. The top 20 most enriched biological process (BP) categories are shown in Figure 8A. The possible driver genes were found to be mainly enriched in “axon guidance,” “cell chemotaxis,” “stem cell differentiation,” and “fat cell differentiation.” The KEGG pathway enrichment analysis revealed that the integrated possible driver genes are enriched in the “MAPK signaling pathway,” “microRNAs in cancer,” “cell cycle,” “regulation of actin cytoskeleton,” and “tyrosine metabolism” (Figure 8B). In summary, these genes related or specific to HCC were found to be significantly correlated with tumor progression, which further highlights their clinical implications.

## Discussion

HCC is an aggressive malignancy, the sixth most common malignancy and the fourth leading cause of cancer-related death worldwide (Llovet et al., 2008). Although advances in treatment strategies have been achieved, no effective molecular targeted therapy has been successfully validated (Lu et al., 2020). HCC is a heterogeneous tumor, and its occurrence, and development is a complex process involving multiple omics changes. This comprehensive multiomics analysis of HCC has broadened our knowledge of the molecular events related to this fatal malignancy (Ress et al., 2015). Herein, we separately analyzed the genome, transcriptome, and methylome of HCC samples and provided new insights for the molecular-level analysis of HCC. In this study, we performed an integrative bioinformatics analysis using a publicly available dataset to emphasize the degree of HCC heterogeneity at the gene expression, mutational, methylation, and CNV levels, and correlated our analysis with clinical features. In the study of Matthew et al., they identified 299 mutation driver genes in the PanCancer data from TCGA (Bailey et al., 2018). Similar to our study, they also found that TP53, ARID1A, PTEN, and other genes were significantly mutated in HCC samples. In another study, the authors used data from 20 cancer types in TCGA to identify methylation driver genes and expression driver genes (Youn et al., 2018), and there is some overlap with our results. Therefore, these results can be cross-validated, further confirming the reliability of our research. After identifying SMGs, we obtained the expression of SMGs in HCC and normal liver tissues in Human Protein Atlas database (https://www.proteinatlas.org/). According to an immunohistochemical staining analysis, five and four SMGs were upregulated (Figure 3B) and downregulated in HCC, respectively, compared with the levels in normal tissues (Figure 3C). In addition, no differences in the expression levels of the three SMGs were found between normal and HCC tissues (Figure 3D).

In total, we identified of 93 possible driver genes in HCC, and these genes might show comprehensive diagnosis and prognostic value for HCC patients. Among the 93 possible driver genes, CDKN2A and CKS1B are worthy of our attention because these are driven by both mutations and CNVs. Somatic mutations in CDKN2A are common in the majority of human cancers. CDKN2A encodes two proteins, namely, the INK4 family members p16 and p14arf (Bartsch et al., 2002), and both of these proteins act as tumor suppressors by regulating the cell cycle. Zhou et al. (2018) indicated that CDKN2A promoter methylation is associated with an increased risk of HCC and plays a crucial role in the process of HCC, which indicates that it has potential value as a triage marker. CKS1B proteins play principal roles in cell cycle regulation. Huang et al. (2010) found that CKS1B overexpression in HCC implicates clinical aggressiveness. The scientific findings of our study further support those obtained in previous studies. The specific mechanisms of the roles of CDKN2A and CKS1B in HCC are worthy of further experimental exploration.

GO enrichment and functional pathway analyses of 93 possible driver genes showed that these genes are mainly involved in “stem cell differentiation,” “cell cycle,” “microRNAs in cancer,” and “MAPK signaling pathway.” These biological processes and KEGG pathways are closely related to the proliferation and apoptosis of cancer cells. Therefore, these results might indicate that the identified possible driver genes are involved in the regulation of HCC growth and are closely related to the occurrence and development of HCC.

Our study has some limitations. First, our research is limited to computer simulations, and thus, our findings should be validated and extended through laboratory experiments. Although our research identified possible driver genes that play important roles in HCC, further experiments are needed to confirm the associated molecular processes. More research is needed to further elucidate the function of the identified possible driver genes, the underlying mechanisms associated with HCC progression, and their potential applications in disease diagnosis and prognosis.

## Conclusion

In conclusion, our study separately analyzed data on the genome, transcriptome, and methylome of HCC samples and constructed a comprehensive multiomics map of HCC. Based on these findings, we believe that the identified possible driver genes play an important role in HCC. In addition, we identified a total of 93 possible HCC driver genes that play an important role in HCC and have prognostic value.

## Data Availability Statement

Publicly available datasets were analyzed in this study. This data can be found here: TCGA LIHC.

## Author Contributions

ZL, YL, and RL: study conception, design, and drafting of the manuscript. XG, RM, XP, and JY: acquisition of data. ZL, RM, XG, YL, and XP: analysis and interpretation of data. JY and RL: critical revision.

## Conflict of Interest

The authors declare that the research was conducted in the absence of any commercial or financial relationships that could be construed as a potential conflict of interest.

## Supplementary Material

Table S1 Tumor ploidy estimated.

Table S2 Epigenetically silenced genes.

Table S3 Differentially expressed mRNA.

Table S4 Differentially expressed miRNA.

Table S5 The scoring table of differentially expressed genes.
